# PD19 Next Generation Methods – Assessment Of Digital Health Applications In Germany

**DOI:** 10.1017/S0266462325101542

**Published:** 2025-12-29

**Authors:** Peter Kolominsky-Rabas, Nikolas Dietzel

## Abstract

**Introduction:**

For the first time, worldwide digital health applications (DiGA) have been reimbursed by the German Statutory Health Insurance. For full reimbursement, the manufacturers must provide scientific evidence based on efficacy studies. This study aimed to assess the methodological quality of efficacy studies for permanently listed and reimbursed DiGA in the categories of ‘nervous system’ and ‘psyche’ within the DiGA register.

**Methods:**

DiGA in the categories of “nervous system” and “psyche” were selected from the governmental DIGA platform. The methodological quality of the studies was assessed using the revised Cochrane risk-of-bias tool for randomized trials. The risk of bias was assessed for the primary endpoint of each study according to an intention-to-treat analysis.

**Results:**

Six DiGA were assessed for their methodological quality. Randomized controlled trials were conducted for all six DiGA that showed a high risk of bias, which was mainly due to a lack of blinding in the studies. In addition, dropouts were significantly higher in the intervention group than in the control group in most studies. For most of the DiGA, no published study protocol was available in advance, so an analysis of potential selective choice of the evaluation methodology was not possible.

**Conclusions:**

The results revealed the low quality of supporting evidence for DiGA recently introduced in Germany, mainly due to methodological flaws, that is, a high potential for bias. Studies of DiGA should be blinded by comparing DiGA with a sham application to reduce the risk of bias. It should be emphasized that manufacturers submitted randomized controlled trials to prove the medical benefit of the DiGA investigated.

